# Fostering the social and physical challenges of female recreational or professional athletes in pregnancy and postpartum

**DOI:** 10.1186/s12884-024-06312-6

**Published:** 2024-02-13

**Authors:** Rita Santos-Rocha, Anna Szumilewicz

**Affiliations:** 1 ESDRM-Sport Sciences School of Rio Maior-Santarem Polytechnic University, Rio Maior, Portugal; 2 SPRINT-Sport Physical Activity and Health Research & Innovation Center , Santarem Polytechnic University, Rio Maior, Portugal; 3 Gdansk University of Physical Education and Sport, Gdansk, Poland

## Abstract

Combining pregnancy and parenthood with sporting activities or a professional athletic career can be challenging. The objective of this Collection is to gain a deeper understanding of the effects of pregnancy and postpartum on female athletes, both recreational and professional, in order to improve their health and fitness outcomes and support their continued success in sports.

## Editorial

The most active period in the sports career of a female professional athlete often coincides with her best reproductive years. Therefore, it frequently becomes necessary to reconcile the expectations and objectives she will have in both these areas. A prolonged sports career requires an adaptation of the training plan allowing the athlete to successfully compete during pregnancy. The training regime must be safe, both for the mother and the fetus, support the birth of a healthy child, and finally return to full competitive activity after birth. Even if the woman is not a professional athlete, but follows a high-volume, high-intensity training program, combining pregnancy and parenthood with athletic activities can be challenging.

Pregnant and postpartum athletes face additional challenges due to uncertainties regarding the normal changes of the body and maintaining a proper healthy lifestyle, the adjustments of their training program, the safety and effectiveness of exercising, and whether they will manage to return to their previous performance and competition level postpartum [1]. Furthermore, professional athletes might be often uncertain whether they will lose their sponsorship or other financial support if they get pregnant and take a long time to get back to a successful sporting career after pregnancy. More female than male athletes decide to retire from their sporting career when they are becoming parents, contributing to gender inequality in sports.

Education is a key dimension for women’s empowerment and health. Moreover, pregnancy is considered to be a “teachable moment” to promote women’s health through the adoption of an active lifestyle. Yet, evidence shows that most pregnant women do not receive proper guidance from public healthcare systems to be physically active [2]. Inactive women are not supported enough to become more active during pregnancy, and exercising women, including pregnant athletes, are often discouraged from remaining active. Moreover, athletes are not satisfied with advice related to strength training and nutrition during pregnancy [3]. This may be because healthcare professionals and sport and exercise professionals do not have adequate knowledge on how and what exercise and lifestyle adaptations they can best recommend to female athletes to enable them to optimize their health and maintain fitness. Sports Medicine doctors, fitness instructors and sport coaches must understand the physiologic and biomechanical factors that are unique to female athletes, and pregnancy in particular, in order to tailor injury prevention programs and optimize their athletic performance [4]. Although current international guidelines on exercise during pregnancy have been updated for sedentary and active pregnant women, they lack contents regarding female athletes [5]. Even the recommendations published under affiliation of the International Olympic Committee lack information on planning and implementing the training process at a high sports level [6, 7]. There is also a lack of high-quality research in this special population [8].

Based on the available literature, the question remains whether high-intensity exercise at > 90% of the maximal aerobic capacity or the maximum heart rate is safe for the mother and fetus. Temporary bradycardia and a decreased uterine artery pulsatility index were observed in fetuses when their mothers exercised above the exertion level of up to 90% maximal aerobic capacity [9]. However, these parameters normalized quickly after the mother interrupted her exercise. In studies analyzing the impact of single exercise sessions and in studies on animal models, the authors did not observe any negative effects for either the mother or the fetus associated with short, high-intensity interval workouts [10]. It should be emphasized that the safe threshold of training intensity is unknown. There is now increasing scientific evidence that well-planned and monitored high-intensity physical activity during pregnancy leads to health benefits. Nevertheless, it is necessary to conduct high-quality research on this topic. For example, whether the brief, frequently repeated bradycardia and reduced placental flow observed during maternal exercise above 90% of maximum exercise capacity may affect the fetus development in long term. There are also many open questions regarding strength training during pregnancy and postpartum. There is little evidence how the changes in blood pressure related to heavy loads can affect the well-being of the pregnant female athlete, the progression of pregnancy and the postpartum recovery. Prevett et al. [11] studied a group of 679 pregnant women who continued weight-lifting training with at least 80% of their maximum load. Their pregnancy complication rates were not different from those of the average population of pregnant women. So far, the upper limit of safe load in strength training has not yet been determined for pregnant and postpartum women.

Therefore, scientific works in this area are extremely valuable, in the forms of biological and social sciences studies, qualitative or case studies, or experimental trials and longitudinal research covering groups of female athletes of particular sports disciplines. These works would be of utmost importance in closing the knowledge gaps of stakeholders and improving the quantity and quality of advice women receive on this topic.

The general objective of this Collection is to gain a deeper understanding of the effects of pregnancy and postpartum on female athletes, both recreational and professional, in order to improve their health and fitness outcomes and support their continued success in sports. Therefore, the relevant stakeholders are not only the women themselves and their babies and families, but also sports and exercise professionals, health care providers, sponsors, sports federations and associations, and policy makers.

The aims of this Collection are aligned with the 2022 Recommendations and action plan from the High-Level Group on Gender Equality in sport “Towards more gender equality in sport”, and with the United Nations’ Sustainable Development Goal number 5 “Achieve gender equality and empower all women and girls”.

## Data Availability

No datasets were generated or analysed during the current study.

## Declarations

### Authors’ information (optional)

See “Meet the Guest Editors” section.
